# Existence of giant mitochondria-containing sheet structures lacking cristae and matrix in the etiolated cotyledon of *Arabidopsis thaliana*

**DOI:** 10.1007/s00709-021-01696-0

**Published:** 2021-08-21

**Authors:** Saki Fukushima, Kae Akita, Tomoko Takagi, Keiko Kobayashi, Nobuko Moritoki, Hajime Sugaya, Shin-ichi Arimura, Haruko Kuroiwa, Tsuneyoshi Kuroiwa, Noriko Nagata

**Affiliations:** 1 Division of Material and Biological Sciences, Graduate School of Science, Japan Women’s University, Bunkyo-ku, Tokyo, Japan; 2 Department of Chemical Biological Sciences, Faculty of Science, Japan Women’s University, Bunkyo-ku, Tokyo, Japan; 3 Laboratory of Electron Microscopy, Japan Women’s University, Bunkyo-ku, Tokyo, Japan; 4 Electron Microscope Laboratory, School of Medicine, Keio University, Shinjuku-ku, Tokyo, Japan; 5 Graduate School of Agricultural and Life Sciences, University of Tokyo, Bunkyo-ku, Tokyo, Japan

**Keywords:** *Arabidopsis*, Cotyledon, Mitochondria, Scanning electron microscopy, Three-dimensional reconstruction

## Abstract

Mitochondria are essential organelles involved in the production and supply of energy in eukaryotic cells. Recently, the use of serial section scanning electron microscopy (S^3^EM) has allowed accurate three-dimensional (3D) reconstructed images of even complex organelle structures. Using this method, ultrathin sections of etiolated cotyledons were observed 4 days after germination of *Arabidopsis thaliana* in the dark, and giant mitochondria were found. To exclude the possibility of chemical fixation artifacts, this study confirmed the presence of giant mitochondria in high-pressure frozen samples. The 3D reconstructed giant mitochondria had a complex structure that included not only the elongated region but also the flattened shape of a disk. It contained the characteristic sheet structure, and the sheet lacked cristae and matrix but consisted of outer and inner membranes. Whether this phenomenon could be observed in living cells was investigated using the transformant with mitochondrial matrix expressing green fluorescent protein. Small globular mitochondria observed in light-treated samples were also represented in etiolated cotyledons. Although no giant mitochondria were observed in light-treated samples, they were found in the dark 3 days after germination and rapidly increased in number on the fourth day. Therefore, giant mitochondria were observed only in dark samples. These findings were supported by electron microscopy results.

**Supplementary Information:**

The online version contains supplementary material available at 10.1007/s00709-021-01696-0.

## Introduction

Mitochondria, bound by a double membrane, are essential organelles for eukaryotes. Their primary function is to generate and supply energy; they are also involved in several functions, such as calcium regulation, oxygen sensing, and apoptosis (Malina et al. 2018). In general, mitochondria are oval, globular, and sausage-like, with an average size of 1 to 2 μm^2^. As known in yeast, mitochondria undergo frequent fission and fusion and sometimes form a tubular network (Lackner 2014). The diameter and length of mitochondria are known to change depending on the cell type and physiological state. In mammalian cells, the suppression of the mitochondrial mitotic factor function has been reported to form an elongated mitochondrial network, suggesting that mitochondrial morphology is maintained due to a balance between fission and fusion (Ban-Ishihara et al. 2013). The structure of mitochondria is intricately associated with their functional status. Giant mitochondria have been reported to occur in variety of human diseases and liver cells of aged humans (Tandler and Hoppel 1986). Free radicals play a crucial role in megamitochondrial formation, followed by apoptosis (Teranishi et al. 1999; Wakabayashi 2002). Murine embryonic fibroblasts treated with a mitochondrial uncoupler showed that more than half of mitochondria presented a ring- or C-shaped morphology (Ding et al. 2012). The loss of mitochondrial membrane potential (MMP) triggers a structural change in mitochondria from a tubular to a globular shape, referred to as mitochondrial fragmentation (Miyazono et al. 2018). Energy-demanding mammalian cells have more elongated mitochondria, whereas energy-rich cells appear to fragment (Liesa and Shirihai 2013). Mitochondria in the axons of highly active neurons contain larger and denser packed lamellar cristae than in less active neurons (Cserep et al. 2018). Mitochondria of neurons are arranged in different sizes depending on their cellular locations (Delgado et al. 2019).

Several studies reported that mitochondria of higher plants also change their shape. The description that giant mitochondria were found in plant cells has been around for a long time (Duckett and Toth 1977). Long and branched mitochondria were observed to surround the nucleus in the shoot apical meristems of *Arabidopsis* (Segui-Simarro et al. 2008). In germinating *Arabidopsis* seeds, mitochondria undergo transient elongation and branching (Paszkiewicz et al. 2017). When *Arabidopsis* plants were kept in the dark for 1 week without sucrose, mitochondria of hypocotyls became longer (Jaipargas et al. 2015). Cold treatment resulted in the transient fragmentation of mitochondria in *Arabidopsis* leaf epidermal cells (Arimura et al. 2017). Low oxygen caused mitochondrial elongation and network formation in suspension-cultured tobacco cells (Van Gestel and Verbelen 2002) and *Arabidopsis* leaf mesophyll cells (Ramonell et al. 2001). The appearance of giant mitochondria was reported in wheat roots in response to the application of respiratory inhibitors (Rakhmatullina et al. 2016). Mitochondria have a characteristic cup shape in egg cells of *Pelargonium zonale* (Kuroiwa et al. 1996). Mitochondria in grapevine leaves changed from elongated and branched structures to enlarged and sparse organelles during senescence (Ruberti et al. 2014). The mechanisms regulating the mitochondrial shape as described above are related to mitochondrial fission. In *Arabidopsis*, the disruption of genes involved in mitochondrial fission leads to a reduced number of mitochondria and the formation of an elongated network (Arimura and Tsutsumi 2002; Arimura 2018; Logan 2006). Mitochondria change their shape depending on the plant tissue or in response to environmental stress. Mitochondria may undergo dynamic structural alterations to meet the changing needs and maintain homeostasis.

Dicotyledon seeds germinated in the dark develop etiolated seedlings characterized by long hypocotyls and smaller cotyledons with a pale yellow color. Plastids in etiolated cotyledons, called etioplasts, contain a special structure known as lattice membranes (Fujii et al. 2018). However, mitochondria have not been sufficiently investigated. This study examined the fine structure and three-dimensional (3D) features of mitochondria in etiolated cotyledons of *Arabidopsis*. Recently, scanning electron microscopy (SEM) has emerged as an important imaging method based on the collection of backscattered electrons (BSE). Serial section SEM (S^3^EM), also known as array tomography, is a novel method to observe ultrathin serial sections on solid substrates. It has become an important tool to visualize the 3D ultrastructure of organelles (Horstmann et al. 2012; Koga et al. 2016; Micheva and Smith 2007). Although fluorescence microscopy can visualize mitochondrial dynamics, visualization of the detailed membrane organization is difficult. Therefore, it is essential to use both fluorescence and electron microscopy to better understand the overall mitochondrial structure, shape, and dynamics within etiolated cotyledons. This study reported giant mitochondria with a complex shape in cotyledons of *Arabidopsis* etiolated seedlings under continuous dark for 4 days.

## Materials and Methods

### Plant materials

*Arabidopsis thaliana* ecotype Columbia was used in this study. To perform fluorescence microscopy, transgenic *Arabidopsis* expressing mitochondrial green fluorescent protein (GFP), whose mitochondrial matrix was visualized by GFP, was used as described previously (Feng et al. 2004). Surface-sterilized seeds were sown on 1/2 MS solid medium (Wako Pure Chemical Industries Ltd., Japan) supplemented with 1.5% (w/v) sucrose and stored at 4°C for more than 2 days. Seeds were preilluminated for 3 h under light and subsequently grown in a chamber at 23°C under continuous dark or light (16:8 h light/dark cycle) for 1, 2, 3, or 4 days. Etiolated seedlings grown under continuous dark were not exposed to light after germination until used in experiments. When fixing, cotyledons were cut off and fixed immediately within 10 min after etiolated seedlings were exposed to light for work. It was confirmed that when etiolated seedlings under dark were moved under light, they quickly greened and followed normal growth.

### Electron microscopy

*Arabidopsis* cotyledons were fixed in 4% (w/v) glutaraldehyde and 4% (w/v) paraformaldehyde, buffered with 50 mM sodium cacodylate at pH 7.0 overnight at 4°C, and washed with the same buffer at 4°C for 4 h. Subsequently, cotyledons were postfixed in 2% (w/v) OsO_4_ with 1.5% (w/v) K_3_[Fe(CN)_6_] in 50 mM cacodylate buffer at 4°C for 2 h. The fixed samples were run through an alcohol series and embedded in Spurr’s resin (Polysciences, Inc., PA, USA). Ultrathin sections (80 nm thick) were cut with a diamond knife (Diatome, Biel, Switzerland) on an ultramicrotome (Ultracut S; Leica, Vienna, Austria).

For SEM, floating serial sections were lifted and mounted on a cover glass (13 mm circle; Matsunami Glass Ind., Ltd., Osaka, Japan). Sections were attached to cover glasses by drying and double-stained with 0.4% uranyl acetate for 10 min and lead citrate solution for 3 min. Subsequently, the cover glass was coated with an osmium coater (Neoc Pro; Meiwafosis Co., Ltd., Tokyo, Japan). Serial sections were observed using SEM with a highly sensitive BSE detector (SU8220; Hitachi, Tokyo, Japan) and an accelerating voltage of 2 kV.

For transmission electron microscopy (TEM), sections were transferred to Formvar-coated grids and double-stained with 4% uranyl acetate for 12 min and lead citrate solution for 3 min. After washing with distilled water, the samples were visualized using TEM (JEM-1400; Jeol, Tokyo, Japan) with an accelerating voltage of 100 kV.

### High-pressure freezing

High-pressure freezing is a method in which living organisms are rapidly chilled to the temperature of liquid nitrogen while simultaneously being exposed to extremely high pressure. Within 10 min after each etiolated seedling grown under continuous dark for 4 days was exposed to light for work, cotyledons were placed on aluminum carriers with 1/2 MS liquid medium containing 1.5% (w/v) sucrose as a cryoprotectant. The samples immediately were cryofixed by high-pressure freezing using an HPM 010 high-pressure freezer (Bal-Tec, Balzers, Liechtenstein). The frozen samples were substituted with 2% (w/v) OsO_4_ in acetone at −80°C for 80 h or more and subsequently at −40°C for 24 h, −20°C for 24 h, and 4°C for 4 h. The samples were gradually replaced from acetone to propylene oxide and then embedded in Spurr’s resin (Polysciences). Ultrathin sections (80 nm thick) were double-stained with 4% (w/v) uranyl acetate for 15 min and lead citrate solution for 7 min. The samples were observed using TEM (JEM-1400; Jeol).

### 3D reconstruction of serial ultrathin section images

Serial ultrathin section images were loaded into Image-Pro 10 (Media Cybernetics, Inc., MD, USA) and automatically and manually aligned. These images were subsequently imported into Adobe Photoshop (Photoshop CC; Adobe Systems, Inc., CA, USA). Mitochondria were manually segmented by tracing their boundary contours. These images were then reimported into Image-Pro, and the 3D reconstructed surface rendering images were created.

### Fluorescence microscopy

A fluorescence microscope (IX-83; Olympus, Tokyo, Japan) equipped with a confocal laser scanning head and control system (FLUOVIEW FV1200; Olympus) was used to acquire confocal images. Maximum-intensity projection images were reconstructed from serial optical sections obtained at 0.5 μm intervals using ImageJ (Schneider et al. 2012).

## Results

### Characteristic shape of mitochondria in cotyledons of etiolated seedlings

**Fig. 1:**
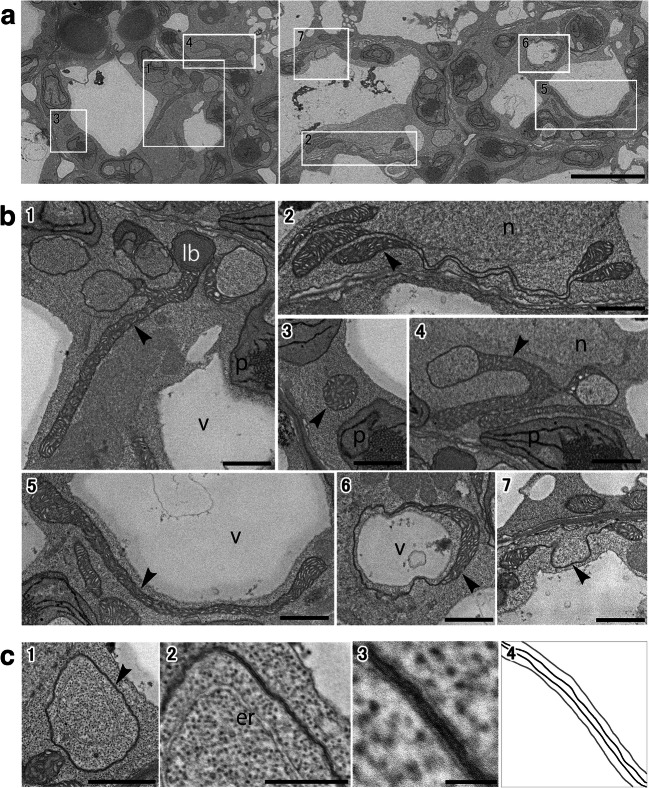
SEM images of ultrathin sections in etiolated cotyledons grown for 4 days in the dark after germination by chemical fixation. **a** Ultrastructural images of mesophyll cells. Squares are placed around those mitochondria intended to be enlarged. **b** High-magnification view of mitochondria (arrowheads). The number of **b** corresponds to each square of **a**. Scale bar, 5 μm (**a**) and 1 μm (**b**). **c** Mitochondrial structure that looks like tubes (arrowhead). The magnification is higher from **c**_**1**_ to **c**_**3**_. **c**_**4**_ is a line drawing of **c**_**3**_. Scale bars, 1 μm (**c**_**1**_), 500 nm (**c**_**2**_), and 10 nm (**c**_**3**_). er, endoplasmic reticulum; lb, lipid body; n, nuclei; p, plastids; v, vacuoles.

Cotyledons of etiolated *Arabidopsis* seedlings grown for 4 days in the continuous dark after germination were observed by the S^3^EM method. Fig. 1 shows a part of an S^3^EM image. Mitochondria were easily identified with typical features, including double membranes and cristae. Mitochondria with giant, unusual, and strange shapes were often found during observation (Fig. 1a).

Several elongated mitochondria were observed, with some having a tubular ring-like connection at the ends (Fig. 1b). The tubular ring contained cytoplasmic components inside and appeared to form a spherical space (Fig. 1b_1_, b_4_, and b_6_). Certain mitochondria appeared connected by elongated tubes (Fig. 1b_2_ and b_7_). Although several such giant mitochondria were found, small and spherical mitochondria were often present (Fig. 1b_3_). However, elongated mitochondria may appear small due to the two-dimensional (2D) image of the section. The mitochondrial tube was extremely thin and lacked cristae. Whether these structures were formed only by the outer membrane of mitochondria or with outer and inner membranes was not determined. Therefore, ring-shaped tubular mitochondrial structures at high magnification were observed (Fig. 1c). Thus, the tube consisted of four membranes (two outer membranes and two inner membranes). In other words, the mitochondrial tube is a highly compressed mitochondrion itself and therefore lacks cristae. The work from fixing to embedding in resin was performed four times for etiolated cotyledons grown for 4 days in the dark. TEM or SEM images of ultrathin sections are provided as supplementary data (Fig. S1). The characteristic mitochondrial structure that looks like tubes was found in all samples.

**Fig. 2:**
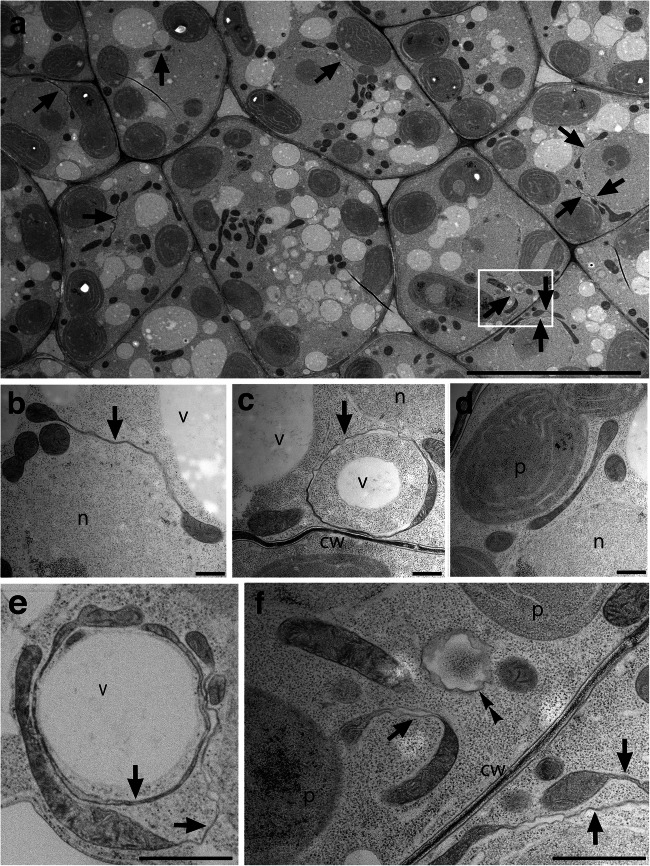
TEM images of ultrathin sections in etiolated cotyledons grown for 4 days in the dark after germination by high-pressure freezing. The mitochondrial structures that look like tubes are indicated by arrows. The double arrowhead indicates the horizontal cross-section of the mitochondrial tube-like structure. **a** Ultrastructural images of mesophyll cells. Squares are the areas to be enlarged in **f**. **b–f** High-magnification view of mitochondria. cw, cell wall. Scale bar, 10 μm (**a**), 500 nm (**b–d**), and 1 μm (**e–f**).

High-pressure freezing is believed to preserve the immediate posture and true shape of all organelles. Therefore, whether giant and strange mitochondria found in etiolated cotyledons could also be observed by freeze fixation was evaluated (Fig. 2). The structure of two mitochondria connected by an elongated tube (like of Fig. 1b_2_ and b_7_) was easily identified, and 10 mitochondrial structures with tubes were found in the field of view of about 10 cells (Fig. 2a, arrows). Unique mitochondrial structures, such as two mitochondria connected by a tube (Fig. 2b), a tubular ring structure (Fig. 2c), an elongated mitochondrion (Fig. 2d), and double tubular mitochondria (Fig. 2e), were also found via cryofixation. In other words, these giant and strange mitochondria found in chemical fixation samples are not artifacts but true figures.

The mitochondrial structure, which looks like a tube in a 2D cross-section, is expected to be a very thin planar structure in 3D. This thin form is termed “sheet.” The double arrowhead in Fig. 2f shows a structure that happens to be a horizontal cross-section of the mitochondrial tube, indicating that it has a thin sheet structure. There were only a few matrix inclusions in the mitochondrial sheet, suggesting that these mitochondria have a unique sheet structure with no cristae and little matrix components.

### 3D reconstruction of mitochondria in etiolated cotyledons

**Fig. 3:**
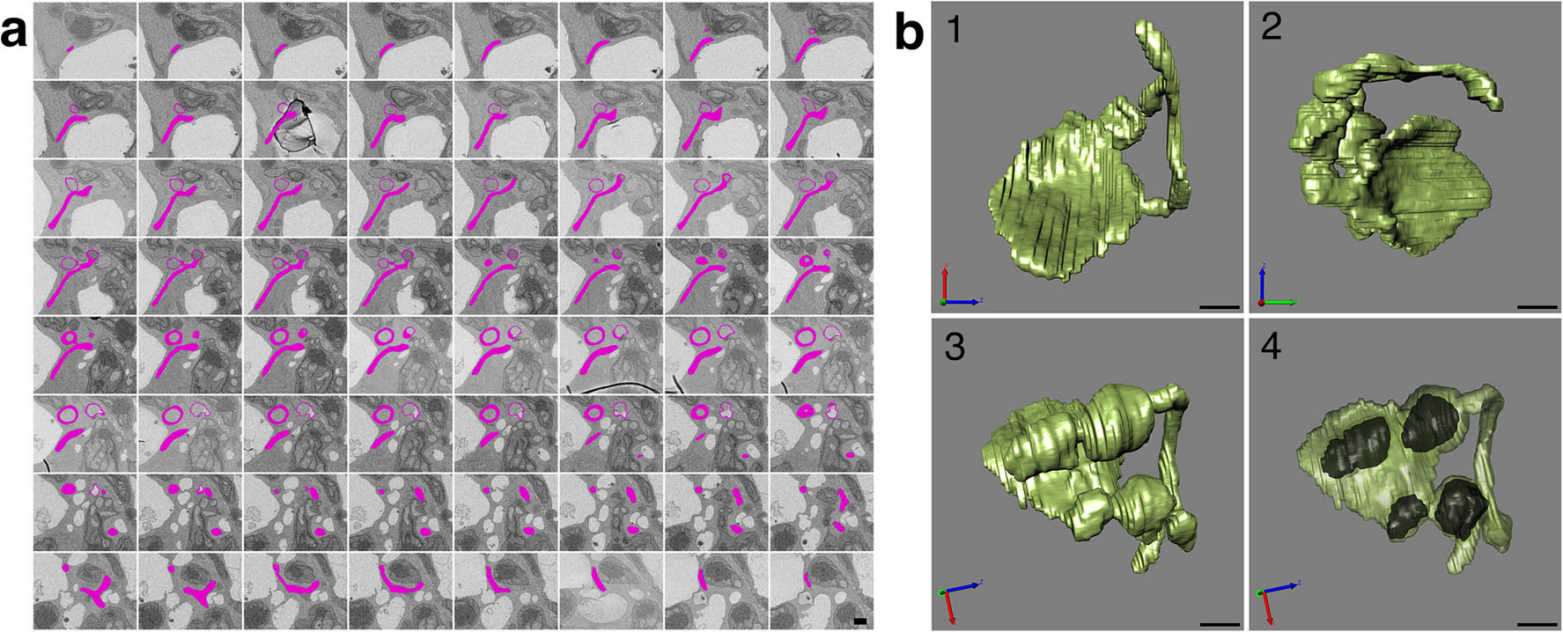
S^3^EM images (**a**) and 3D reconstructed images (**b**) correspond to mitochondria indicated by arrowheads in Fig. **1b**_**1**_. **a** Images consist of a set of 64 serial sections. Target mitochondria are pink. **b** Surface rendering images were obtained from various angles. **3b**_**4**_ is a semitransparent image of **3b**_**3**_, where black areas indicate the cytosolic space. Scale bar, 1 μm.

**Fig. 4:**
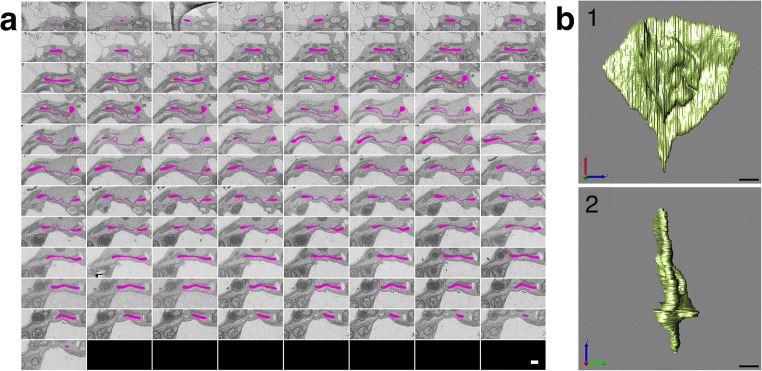
S^3^EM images (**a**) and 3D reconstructed images (**b**) correspond to mitochondria indicated by arrowheads in Fig. **1b**_**2**_. **a** Images consist of a set of 89 serial sections. Target mitochondria are pink. **b** Surface rendering images were obtained from various angles. Scale bar, 1 μm.

**Fig. 5:**
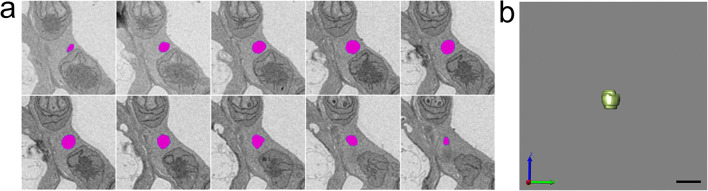
S^3^EM images (**a**) and 3D reconstructed image (**b**) correspond to mitochondria indicated by arrowheads in Fig. **1b**_**3**_. **a** Images consist of a set of 10 serial sections. Target mitochondria are pink. **b** Surface rendering image. Scale bar, 1 μm.

Acquiring high-resolution 3D ultrastructural data is crucial to deepen the understanding of the true mitochondrial shape. Because it was difficult to fix well to the deep part by cryofixation, 3D reconstruction of some mitochondria was performed using S^3^EM in chemical fixation (Figs. 3–5). Fig. 3a shows 64 slices of ultrathin sections of the mitochondrion as indicated by Fig. 1b_1_ (arrowhead). Fig. 3b consists of mitochondrial 3D reconstruction images created from Fig. 3a, obtained from several angles. Similarly, Fig. 4a shows 89 slices, as indicated by Fig. 1b_2_ (arrowhead), and Fig. 5a shows 10 slices of that in Fig. 1b_3_. Figs. 4b and 5b are mitochondrial 3D reconstruction images created from Figs. 4a and 5a, respectively. Movies S1 to S3 provide rotating animations of mitochondrial 3D images as shown in Figs. 3b, 4b, and 5b, respectively.

What was recognized as a separate mitochondrion on the section was often actually connected. The mitochondrion in Fig. 3 had a complex structure that included not only the elongated region but also the flattened shape of a disk and the very thin form that we call a sheet. The sheet lacked cristae, had little matrix, and consisted of the outer and inner membranes of the mitochondrion, such as in Fig. 1c. These structures offered spherical cytosolic space (Fig. 3b_4_, black area). Interestingly, there were no orifices in this spherical space. This appeared to not provide a pathway connecting the internal space to the cytoplasm. The complex and giant mitochondrion with multiple and several interconnected structures, such as those shown in Fig. 4, was frequently confirmed by 3D analysis. The delimited space was not always created within giant mitochondria. Moreover, mitochondria with a large opening in a vase-like shape were also frequently found.

Although the structure in which two mitochondria appeared to be connected by a tube was often observed in a 2D section (Figs. 1 and 2), 3D reconstruction revealed these tubes to be sheets (Fig. 4). The size of the mitochondrion was large, and the sheet area occupied most of the mitochondrion. This giant mitochondrion was expected to have lower amounts of inner membrane and matrix compared to the outer membrane than normal mitochondria.

In addition to the large mitochondria, small, spherical, common mitochondria were frequently found (Fig. 5). The internal structure, such as cristae, of small mitochondria was similar to giant mitochondria, except for the sheet. Small mitochondria do not exist in specific tissues or cells but coexist randomly with large mitochondria.

### Observation of giant mitochondria in living cells by fluorescence microscopy

**Fig. 6:**
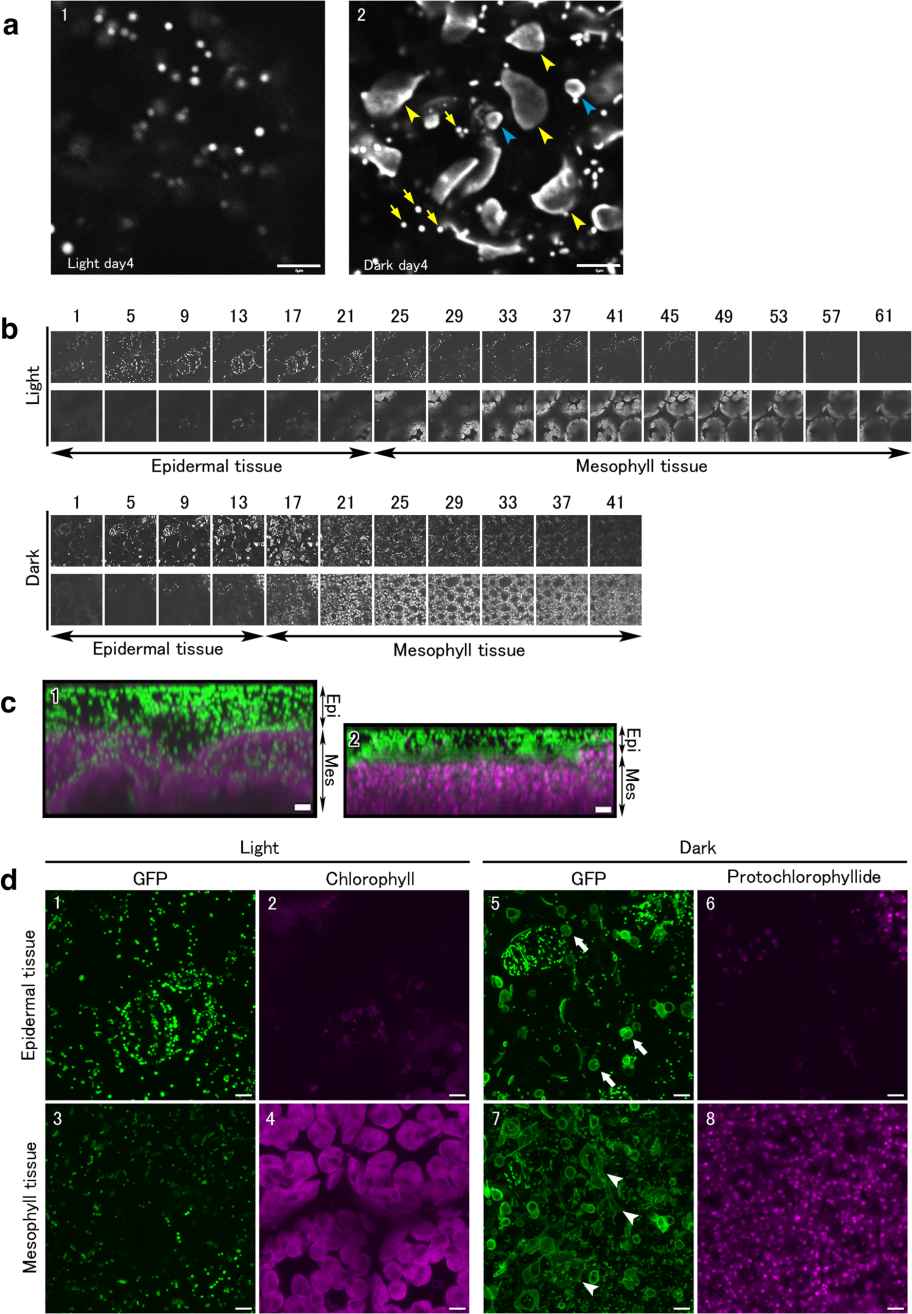
Fluorescence images of mitochondrial GFP in cotyledons. **a** A single optical section image of the mesophyll tissues of the cotyledon at 4 days after germination in the light (**a**_**1**_) or dark (**a**_**2**_). Yellow arrowheads, blue arrowheads, and yellow arrows indicate giant mitochondria, small donut-shaped mitochondria, and small spherical mitochondria in the dark, respectively. **b** Z-stack images from the epidermal tissue side toward the mesophyll tissue side. Top row, cotyledon in the light; bottom row, cotyledon in the dark. The top of each column shows mitochondrial GFP images, and the bottom shows autofluorescence images of chlorophyll or protochlorophyllide. Section numbers from a set of 64 sheets in the light and 44 sheets in the dark are shown outside each column. **c** Images of the Z-stack projection observed from the vertical section of cotyledons in the light (**c**_**1**_) or dark (**c**_**2**_). Mitochondrial GFP is shown as green, and autofluorescence of chlorophyll or protochlorophyllide is shown as magenta. **d** Maximum-intensity projection images of epidermal tissues (**d**_**1**_, **d**_**2**_, **d**_**5**_, **d**_**6**_) or mesophyll tissues (**d**_**3**_, **d**_**4**_, **d**_**7**_, **d**_**8**_) in the light (**d**_**1**_–**d**_**4**_) or dark (**d**_**5**_–**d**_**8**_). Mitochondrial GFP (**d**_**1**_, **d**_**3**_, **d**_**5**_, **d**_**7**_) and autofluorescence of chlorophyll (**d**_**2**_, **d**_**4**_) or protochlorophyllide (**d**_**6**_, **d**_**8**_) are shown. Scale bar, 5 μm.

Whether giant mitochondria were observed in living cells using plants whose mitochondrial matrices were visualized by GFP was investigated. Mesophyll tissues of cotyledons at 4 days under light or dark were observed as a single optical section image (Fig. 6a). Mitochondria under light showed a spherical shape of about 1 μm, whereas those under dark had a conspicuous donut shape or atypical giant mitochondria exceeding 5 μm (yellow arrowheads). In the dark, small donut-shaped mitochondria (blue arrowheads) were observed as well as small spherical or sausage-shaped mitochondria similar to those in the light (yellow arrows). These fluorescence microscopy results were consistent with electron microscopy, where both giant and small mitochondria were present in the dark, demonstrating that no giant mitochondria were observed in the light.

Because a confocal laser scanning microscope can easily observe a wider area than an electron microscope, epidermal and mesophyll cells of cotyledons were distinctly observed. In addition to the cell shape, autofluorescence of chlorophyll or protochlorophyllide (a precursor of chlorophyll) was used as a standard to determine whether the observed region was mesophyll tissue. Z-stack images were acquired from the epidermal tissue side toward the mesophyll tissue side. The snapshots selected from the Z-stack images of 64 sheets in the light and 44 sheets in the dark were shown for every three images (Fig. 6b, Movies S4 and S5). Fig 6c is an image of the Z-stack projection observed from the vertical section. In addition to mitochondrial GFP, autofluorescence images of chlorophyll or protochlorophyllide were acquired, as shown by the maximum-intensity projection images (Fig. 6d). Under dark conditions, donut-shaped mitochondria of epidermal tissue were small and shown as simple circles (Fig. 6d, arrows), whereas donut-shaped mitochondria of mesophyll tissue were large, and a few were above 10 μm (Fig. 6d, arrowheads). Mitochondria under light showed a small and globular shape in both epidermal and mesophyll tissue.

### Examination of when giant mitochondria are formed

Giant mitochondria were examined after germination in the dark. *Arabidopsis* seedlings were grown under dark or light for 1, 2, 3, or 4 days and observed.

**Fig. 7:**
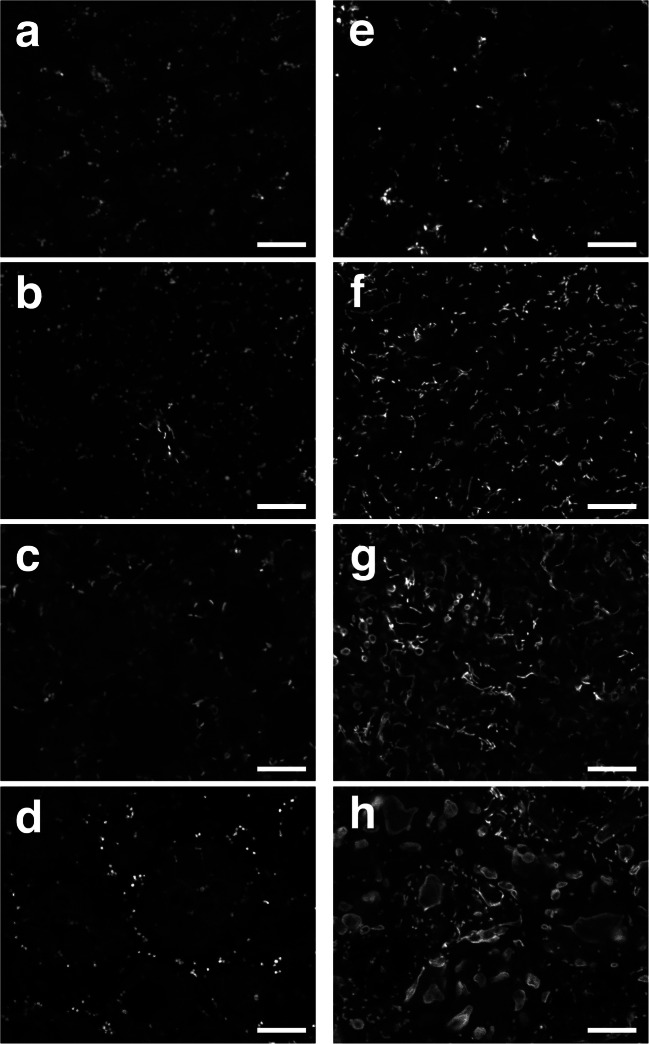
Fluorescence images of mitochondrial GFP in cotyledons of seedlings grown in the light or dark. **a–d** Images of seedlings grown for 1 day (**a**), 2 days (**b**), 3 days (**c**), and 4 days (**d**) after germination in the light. **e–h** Images of seedlings grown for 1 day (**e**), 2 days (**f**), 3 days (**g**), and 4 days (**h**) after germination in the dark. Scale bar, 10 μm.

Fig. 7 shows fluorescence microscopy images of mitochondrial GFP. In the light, no giant mitochondria were observed from 1 to 4 days. In the dark, more sausage-like mitochondria were observed after 2 days instead of spherical mitochondria. Small donut-shaped mitochondria were observed 3 days after germination. Donut-shaped mitochondria 3 days after germination were smaller and fewer in number than those 4 days after germination. Giant mitochondria were estimated to rapidly develop between 3 and 4 days after germination in the dark.

**Fig. 8:**
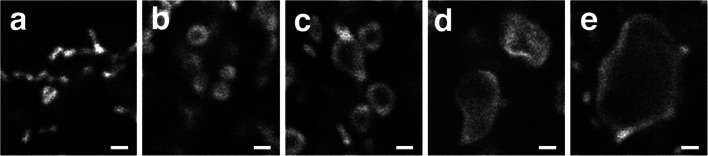
Fluorescence images of mitochondrial GFP showing the formation process of giant mitochondria. **a–e** Expected giant mitochondrial formation process. Images of seedlings grown for 2 days (**a**), 3 days (**b** and **c**), and 4 days (**d** and **e**) after germination in the dark. Scale bar, 1 μm.

The images from 3 to 4 days after germination in the dark appeared to contain various stages until the formation of giant mitochondria. Therefore, these images were arranged in the estimated order of formation stages (Fig. 8). Initially, mitochondria are sausage-shaped (Fig. 8a). Then, it takes on a small donut shape, but it may be flat like an erythrocyte because GFP fluorescence is still seen at the center of the donut (Fig. 8b). It gradually increases in size while remaining donut-shaped (Fig. 8c), and the area of the sheet structure expands (Fig. 8d). Eventually, it transforms into a giant mitochondrion with a thin sheet structure occupying most of the area (Fig. 8e).

**Fig. 9:**
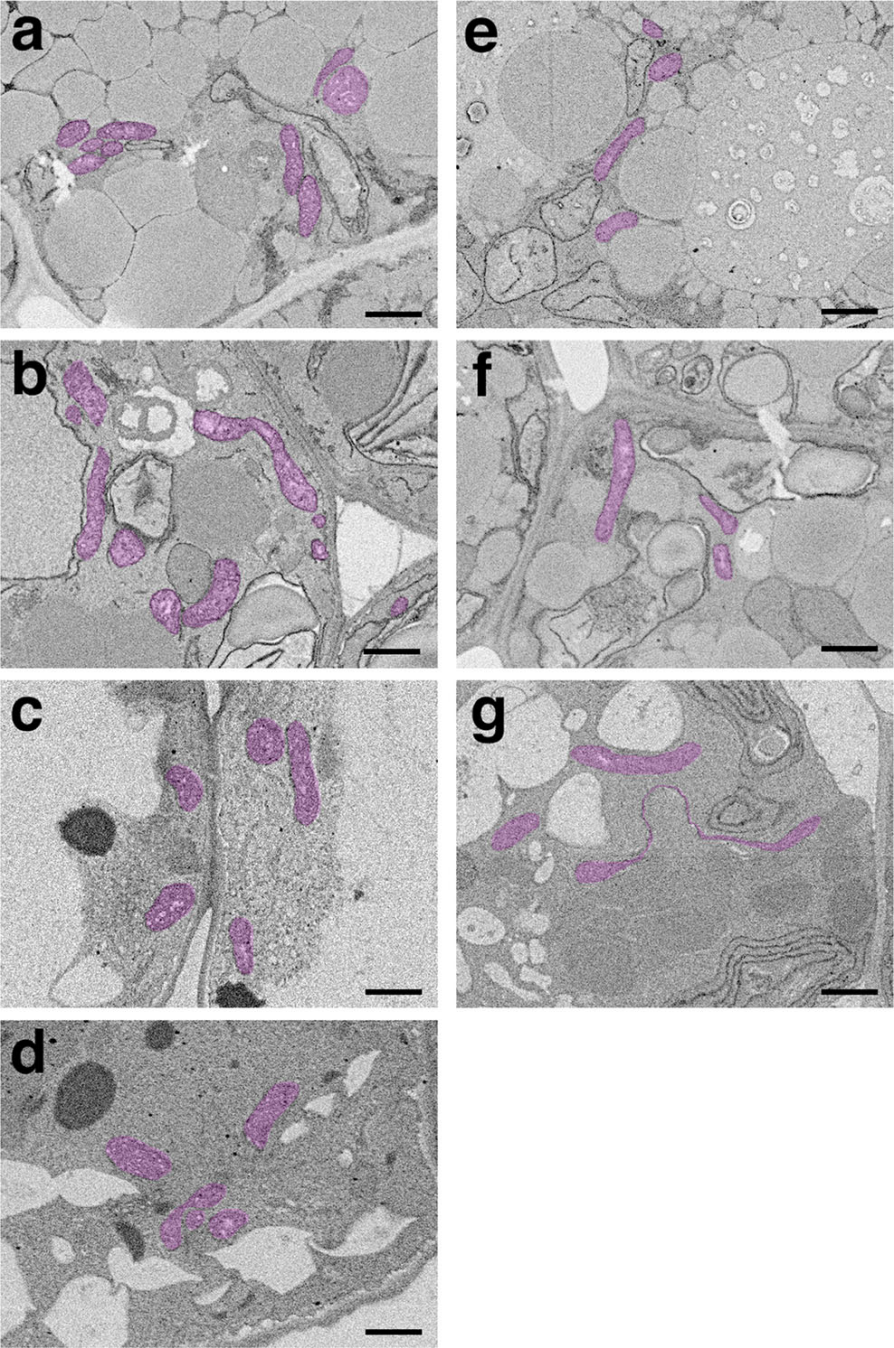
SEM images of ultrathin sections in cotyledons of seedlings grown in the light or dark. **a–d** SEM images of seedlings grown for 1 day (**a**), 2 days (**b**), 3 days (**c**), and 4 days (**d**) after germination in the light. **e–g** SEM images of seedlings grown for 1 day (**e**), 2 days (**f**), and 3 days (**g**) after germination in the dark. The pink area represents mitochondria. Scale bar, 1 μm.

Subsequently, ultrathin sections were observed using SEM (Fig. 9). Mitochondria with the characteristic sheet structure were observed only on the third and fourth days in the dark. Moreover, a few mitochondria with sheets were observed within 3 days, but they were all small (Fig. 9g). Fluorescence microscopy results (Fig. 7) were supported by electron microscopy results (Fig. 9).

## Discussion

Germination and growth of *Arabidopsis* seeds in the dark revealed etiolated cotyledons that contained a considerable number of giant and complex mitochondria that appeared on the fourth day. The unique feature is that giant mitochondria have an extremely large sheet area with few cristae and matrices, where the inner membranes were close to each other (Fig. 1c). The sheet rounds into a sphere and creates a space filled with the cytoplasm (Fig. 3) or spreads out greatly to form a thin shape (Fig. 4). Such mitochondria have a large outer membrane than the inner membrane and matrix. The reason for this structure could be that the outer membrane increases the surface area for substance exchange and communication with the cytoplasm. Moreover, it could be too late to increase the volume of the matrix, resulting in a sheet structure. In any case, the phenomenon of having such a wide sheet structure and forming giant mitochondria, sometimes exceeding 10 μm, is surprising.

In this study, several mitochondria observed under fluorescence microscopy had a donut and elongated shape than under electron microscopy. The reason could be the detection of GFP in the matrix. The majority of mitochondria, which looked like giant donuts under fluorescence, could actually be covered with sheets. However, these cannot be observed well because there was little matrix in the sheets. Even if giant mitochondria have a barrel-like structure and are filled with cytoplasm, they may look like having a donut shape under fluorescence microscopy. Moreover, despite the wide sheet structure of mitochondria, these may look like a donut. It is difficult to capture the true form without electron microscopy. However, the conventional serial section TEM method is unsuitable for observing large areas. In addition, it was not possible to construct an accurate 3D image due to technical difficulties and a large amount of distortion and loss of sections. In contrast, the S^3^EM method used in this study is an excellent new technique that overcomes these shortcomings (Koga et al. 2016). It is easier to identify the unique structure of several organelles, such as giant mitochondria, using this new technique.

A phenomenon called mitochondrial fragmentation has been discovered for a long time, and it seems that it has recently attracted attention in relation to autophagy (Zorov et al. 2019). The loss of MMP changes the mitochondrial shape from a tubular to globular shape that is swelled or ring/donut shape (Miyazono et al. 2018). Although this fragmentation appears to be the opposite of giant mitochondrial formation, the small mitochondrial donut form that results from fragmentation is similar in shape to the giant mitochondrial donut form observed. In particular, small donut-shaped mitochondria found after 3 days in the dark, the early stage of the giant mitochondrial formation process (Figs. 7 and 8), are very similar. The mitochondrial formation in this study may have a mechanism similar to this fragmentation. A previous study exhibited that mitochondrial DNA defects increase mitochondrial nanotunnels defined as a connecting membrane across nonadjacent mitochondria (Vincent et al. 2019). Furthermore, mitochondria had tubular protrusions of the outer membrane with little or no matrix, known as mitochondrial outer membrane protrusions (MOPs). MOPs frequently form a bridge between two mitochondria and increase in the number of senescent leaves after dark treatment (Yamashita et al. 2016a, 2016b). These nanotunnels or MOPs may contribute to the formation of giant mitochondria—a combination of multiple structures shown in Fig. 3.

Previously discovered aberrant mitochondria are generally thought to develop under stress. Giant mitochondria found here are in healthy etiolated seedlings in the dark. This is a normal developmental process from underground to aboveground, although the lack of light is a serious situation for plants. The formation of giant mitochondria may have been triggered by stress (i.e., darkness) in the early stages (Figs. 7g and 8b and c), but they may have continued to expand their surface area as a way to overcome stress (Fig. 7h and 8d and e). Why are giant mitochondria made in the etiolated cotyledon? Plant mitochondrial dynamics may change as early events during the induction of programmed cell death (Logan 2010). Earlier studies have indicated that giant mitochondria are formed due to hypoxia (Ramonell et al. 2001; Van Gestel and Verbelen 2002) or in response to reduced cytosolic sugar levels (Jaipargas et al. 2015). The seedlings used in these experiments were grown in a medium containing sucrose. The finding that mitochondria become enlarged in the dark may be the result of nonphotosynthetic hypoxia rather than effects of low sugar levels. The exposure of pulmonary artery endothelial cells to hypoxia caused the perinuclear clustering of mitochondria to govern the local concentrations of second messengers, such as reactive oxygen species (Al-Mehdi et al. 2012). Thin and flat giant mitochondria were often present near the nuclei (Figs. 1b_2_ and b_4_ and 2b; Fig. S1a–S1c). Perhaps the perinuclear clustering of mitochondria in endothelial cells and giant mitochondria around nuclei in etiolated cotyledons might capture a similar phenomenon. It is possible that prolonged exposure to dark has led to severe hypoxia and even more accumulation (giantization) than clustering.

Recently, it has been recognized that the interaction between organelles is ubiquitous and the membrane contact site functions as a major pathway for intracellular transport between organelles (Valm et al. 2017). Furthermore, close associations between mitochondria and endoplasmic reticulum (ER) have been reported in yeast, mammalian cells, and plant cells, suggesting a correlation between mitochondrial pleomorphy and the ER (Friedman et al. 2011; Jaipargas et al. 2015). In this study, lipid bodies (Fig. 1b_1_), vacuoles (Figs. 1b_6_ and 2c), or endoplasmic reticulum (Fig. 1c_2_) were captured in the closed cytoplasmic space created by the mitochondrial sheet in giant mitochondria. It is unclear whether enclosed organelles were actively or passively engulfed and contacted by mitochondria. However, mitochondrial sheets in this study may facilitate some communication with other organelles. The engulfed organelles vary and therefore appear nonselective. With the formation of giant mitochondria, one more question remains: that not all mitochondria are giant. The presence of several small spherical mitochondria makes it hard to believe that they are in the process of giantization. Small and giant mitochondria coexist in the same cell. Why mitochondria with these shapes are formed awaits further investigation.

## Supplementary information

Fig. S1 TEM or SEM images of ultrathin sections in etiolated cotyledons grown for 4 days in the dark after germination. The work from fixing to embedding to resin was performed four times. **a–c** TEM images of samples were fixed on the same day, but each image is from different plants. **d–f** SEM images of samples were fixed on different days. The characteristic mitochondrial structure that looks like tubes (arrows) was found in all samples. (PNG 2880 kb)

High Resolution Image (TIF 3649 kb)

Movie 1. 3D reconstruction of a mitochondria created from Fig. 3 (MP4). (MP4 3464 kb)

Movie 2. 3D reconstruction of a mitochondria created from Fig. 4 (MP4). (MP4 5956 kb)

Movie 3. 3D reconstruction of a mitochondria created from Fig. 5 (MP4). (MP4 1269 kb)

Movie 4. Z stack images of mitochondrial GFP in the cotyledon in the light (MP4). (MP4 2673 kb)

Movie 5. Z stack images of mitochondrial GFP in the cotyledon in the dark (MP4). (MP4 7211 kb)
