# Supplementary figures and images for: Existence of giant mitochondria-containing sheet structures lacking cristae and matrix in the etiolated cotyledon of Arabidopsis thaliana
Source: Protoplasma. 2021 Aug 21;259(3):731–42. doi: 10.1007/s00709-021-01696-0 (PMC9010340; doi:10.1007/s00709-021-01696-0)

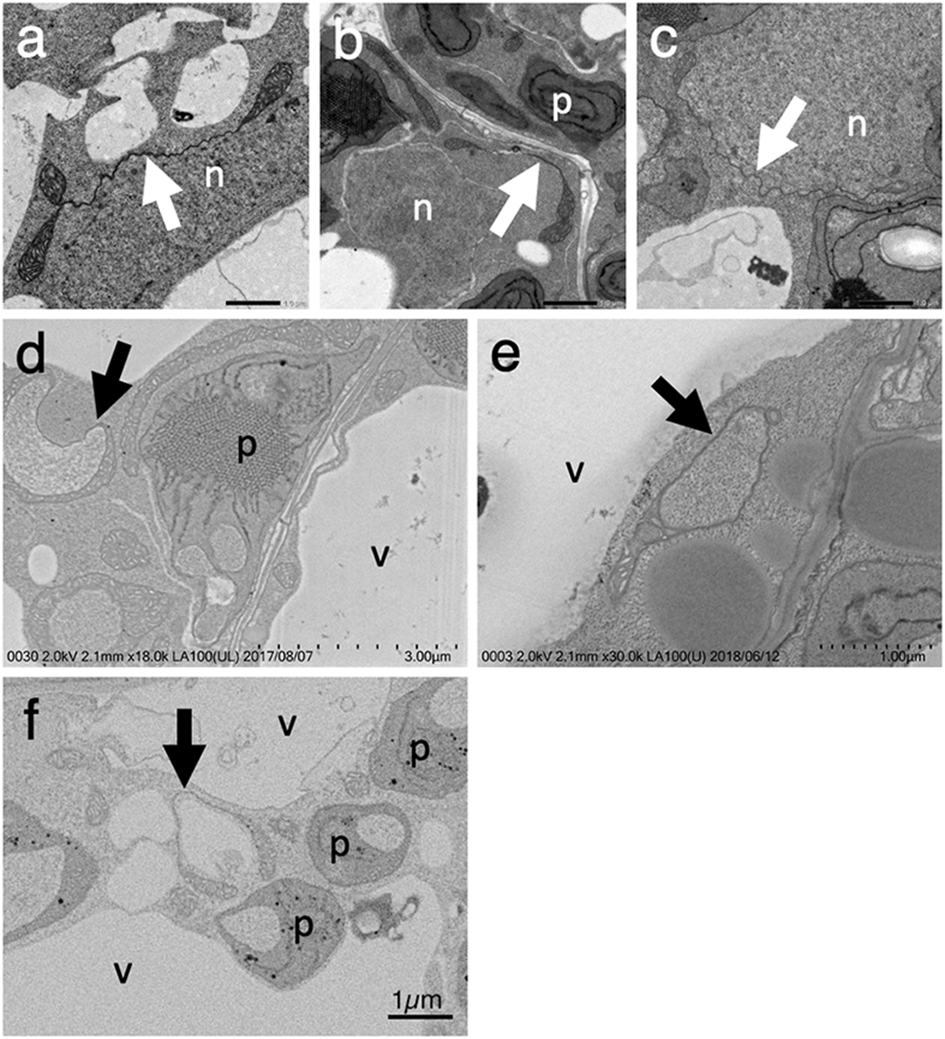

Supplement: Supplementary file 1 — TEM or SEM images of ultrathin sections in etiolated cotyledons grown for 4 days in the dark after germination. The work from fixing to embedding to resin was performed four times. a–c TEM images of samples were fixed on the same day, but each image is from different plants. d–f SEM images of samples were fixed on different days. The characteristic mitochondrial structure that looks like tubes (arrows) was found in all samples. (PNG 2880 kb) [file 709_2021_1696_Fig1_ESM.png]

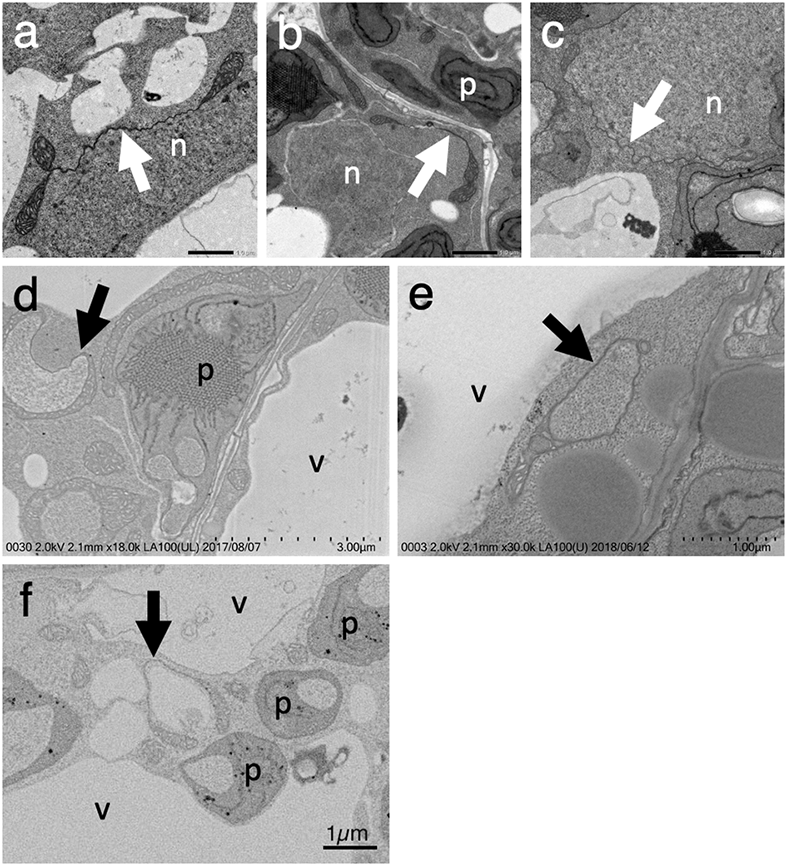

Supplement: Supplementary file 2 — High Resolution Image (TIF 3649 kb) [file 709_2021_1696_MOESM1_ESM.tif]
